# Water-Assisted Electrosynthesis of a Lithium–Aluminum Intermetallic from a Lithium Chloride-Ionic Liquid Melt

**DOI:** 10.1021/acselectrochem.4c00134

**Published:** 2025-01-17

**Authors:** Fabrizio Bernini, Giovanni Bertoni, Adele Mucci, Andrea Marchetti, Daniele Malferrari, Gian Carlo Gazzadi, Marco Ricci, Sergio Marras, Remo Proietti Zaccaria, Enzo Rotunno, Alessio Nicolini, Nassima Yamini, Andrea Cornia, Marco Borsari, Andrea Paolella

**Affiliations:** † Dipartimento di Scienze Chimiche e Geologiche e UdR INSTM, Università degli Studi di Modena e Reggio Emilia, Via G. Campi 103, 41125 Modena, Italy; ‡ CNR − Istituto Nanoscienze, Via G. Campi 213/A, 41125 Modena, Italy; § Istituto Italiano di Tecnologia, Via Morego 30, 16163 Genova, Italy

**Keywords:** Water, Ionic Liquid, Lithium, Aluminum, Intermetallic

## Abstract

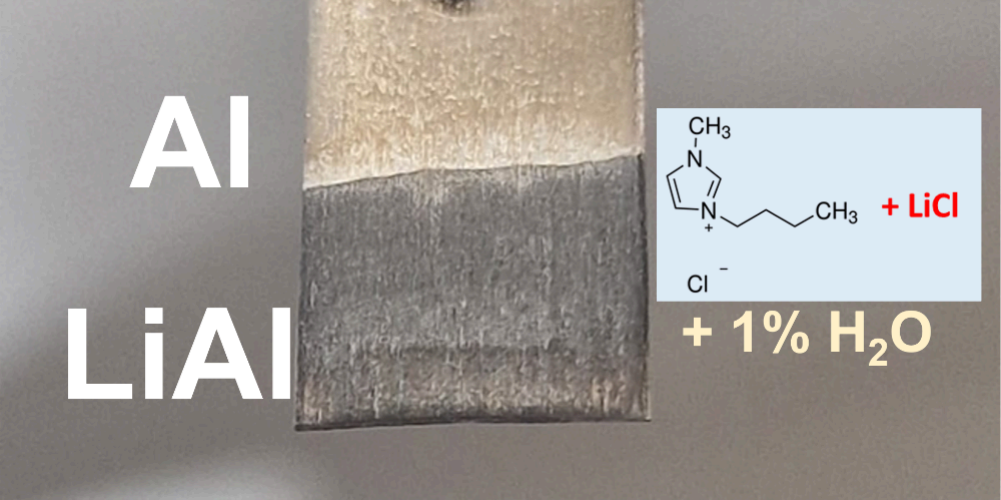

Although water is considered detrimental for Li-ion battery technology, a 1% w/w amount of water in a melt of LiCl in ionic liquid 1-butyl-3-methylimidazolium chloride promotes the reduction of lithium into a LiAl intermetallic along with water oxidation to O_2_ gas. The electrodeposition of an intermetallic layer of several micrometers thickness is demonstrated by combining complementary techniques, such as galvanostatics, X-ray diffraction, electron energy-loss spectroscopy, mass spectrometry, and ^1^H nuclear magnetic resonance. The concentration of water in the ionic liquid is found to be a critical feature, as no Li is deposited when ionic liquid is dried. Our findings highlight an innovative and simple method to produce a LiAl intermetallic by using water and lithium chloride as chemical reagents.

## Introduction

Lithium metal is without a doubt a fundamental resource for humankind.^1^ It finds its main use in organic chemistry as a reductant to produce organolithium compounds,^2^ which are important precursors for pharmaceuticals,^3^ for the Birch reduction,^4^ and in preparing vitamins such as vitamin A.^5^ Since the 1990s, lithium-ion batteries (LIBs) have been widely used as preferred energy storage systems for electric vehicles and portable electronic devices because Li has the lowest electrochemical potential (−3 V vs H^+^/H_2_) and the highest power density per charge among anode materials.^6−8^ The use of Li metal as anode can strongly increase the final energy density of the batteries (up to 450−500 Wh kg^−1^).^9,10^ Industrially, Li metal ingots are generally produced by electro-extraction of molten LiCl-KCl eutectic in the temperature range of 450–500 °C.^11^ The LiCl-KCl eutectic is formed at 353 °C with a molar ratio of 59:41. Since the melting point of Li (180 °C) is lower than the temperature of the reaction bath, the product is formed as a liquid, while chlorine is produced at the anode:
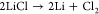


At the end of the redox reaction, Li metal floats on the surface of the LiCl-KCl eutectic and can be easily isolated. Foils are then produced from Li metal ingots mainly by a rolling method. Li is extruded by applying pressure through a hydraulic ram using a fluid to avoid any contamination. At this stage, the Li metal thickness is around 200–400 μm. Rolling allows the further reduction of the thickness down to 10–20 μm, which represents the lowest limit for a self-standing Li metal film obtained via rolling technique. Below this threshold, a current collector is necessary to accomodate a layer of Li metal. A possible method involves the production of Li on copper foil. First, a 450-nm-thick lithiophilic Cu oxide layer is formed on 6 μm Cu foil by heating at 200 °C in the air. Then, it is possible to deposit a 10 μm layer of metallic Li by dipping the Cu foil in a molten Li bath.^12^ Thermal evaporation under a vacuum (*p* = 10^–4^ Torr at 407 °C) can also provide micron-thick layers of Li metal.^13^ This method is already industrially scalable for microbattery application, although the high vacuum systems represent a cost limitation. Other lab-scale vapor-based methods have been developed to deposit thin layers of Li metal^14,15^ such as electron beam vapor deposition, pulsed laser deposition, and sputter deposition. Lithium is also highly valuable as an alloying element:^16^ it infers lightness and mechanical strength to aluminum^17^ and magnesium,^18^ it is used in nuclear fusion technology when alloyed with lead,^19^ and it is alloyed with boron to prepare negative electrodes for thermal batteries.^20^

Aluminum is the second most produced metal in the modern world (58.8 million metric tons). Thanks to its low density (2.7 g cm^–3^), it is used in several fields such as aerospace, automobiles, packaging, and energy applications.^21^ Al is demonstrated to be a promising intermetallic-type (more commonly called alloy-type^22^) active material to produce negative electrodes for LIBs. This metal possesses a significant lithium storage capacity (993 mAh g^–1^) and a low intercalation/deintercalation potential versus Li^+^/Li^0^ (∼0.2–0.3 V),^23,24^ which makes it interesting as a substitute of the lithium–metal electrode. During the lithiation process, Al can form several intermetallic compounds, such as β-LiAl (named LiAl in the following), Li_3_Al_2_, and Li_9_Al_4_.^25,26^ Li–Al intermetallics can be produced by pyrometallurgical (annealing of Li and Al powder in He atmosphere at 360° C) or electrodeposition processes (Li deposition on Al foil in 1 M LiClO_4_-tetrahydrofuran electrolyte).^27^ Prelithiated Al anodes exhibited promising results: for instance, Pan et al.^28^ fabricated a Li_0.8_Al anode by pressing Al and Li foils together and subsequently prepared a Li_0.8_Al//sulfur battery, which was able to deliver a capacity of 1237 mAh g^–1^ after 200 cycles at 0.2C with a high-capacity retention of 93%. Chen et al.^29^ prelithiated an Al foil by electrodeposition method at a current density of 50 μA cm^–2^, achieving a total capacity of 4 mAh. A full cell composed of an electrodeposited LiAl anode and LiFePO_4_ cathode exhibited a capacity of 146 mAh g^–1^ after 1000 cycles at a charge/discharge rates of 1C–1C.

In nonaqueous electrolytes, the presence of water usually represents a detrimental factor for both Li metal and LIBs due to the possibility to generate hydrogen gas at the negative electrode and oxygen gas at the positive electrode. Moreover, water can react with LiPF_6_ salt to produce HF in the electrolyte, along with an insulating LiF layer:
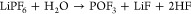
However, Xiong et al.^30^ discovered that adding up to 2000 ppm of H_2_O to the electrolytes had no adverse effect on the performance of LiCoO_2_//graphite pouch cells.

Ionic liquids (ILs) have been explored as solvents in LIBs due to their wide electrochemical stability. They comprise an organic cation (e.g. imidazolium, phosphonium, pyridinium, pyrrolidinium, alkylammonium) and an anion that may be either inorganic (e.g. chloride, bromide) or organic (e.g. triflate, benzoate, sulfacetamide, bis(trifluoromethanesulfonyl)imide, bis(fluorosulfonyl)imide).^31−34^ For instance, 1-butyl-3-methylimidazolium chloride (BMIM-Cl), a popular IL, was tested as a solvent by Jie et al.^35^ to electrodeposit Cu–Sn alloy from dissolved hydrated copper and tin salts. Innocenti et al.^36^ demonstrated that [Al_2_Cl_7_]^−^ is an electroactive species when BMIM-Cl and AlCl_3_ are mixed together. Nguyen et al.^37^ investigated LiCl/[imidazolium]Cl melts as catalysts for the coupling reactions of propylene oxide and CO_2_ and found evidence of polynuclear species [Li_*n*_Cl_*n*+1_]^−^ (*n* = 2–5) along with [LiCl_2_]^−^.

In this work, we demonstrate for the first time that water plays a pivotal role in the Li intermetalizing process on Al metal during the electrolysis of a LiCl/BMIM-Cl melt. A weight percentage of water around 1% w/w can promote the reduction of lithium ions and the formation of LiAl intermetallics by serving as a source of electrons and producing oxygen gas. Rewardingly, the described electrolysis process can be performed at 80 °C, a temperature much lower than that needed for the electro-extraction of molten LiCl-KCl eutectic (450–500 °C). Note that water is unable to decompose the electrodeposited LiAl intermetallic at this concentration, while for substantially higher amounts of water (e.g. 10% w/w), H_2_ formation at the cathode becomes favored over Li intermetalizing.

## Experimental Section

### Sample Preparation

A LiCl/BMIM-Cl solution containing residual water from the IL (H_2_O-LiCl-IL) was prepared in a glove box by mixing 3.48 g (19.9 mmol) of as-received BMIM-Cl and 0.40 g (9.4 mmol) of anhydrous LiCl. The amount of water was quantified at around 1% w/w by ^1^H NMR analysis. The vial was heated at 80 °C and directly used as an electrolytic cell with graphite as a positive electrode and a 100-μm-thick aluminum foil as a negative electrode. A second sample (dry-LiCl-IL) was prepared by heating the above LiCl/BMIM-Cl solution at 120 °C for 40 h under a vacuum to remove residual moisture. In order to confirm the presence of HCl, 100 μL of 1 M aqueous solution of HCl was added to 3.88 g of H_2_O-LiCl-IL. The samples containing 3%, 5%, 8%, and 10% water (w/w) were prepared by adding water to the H_2_O-LiCl-IL sample.

### Electrochemistry

Galvanostatic measurement was performed using a potentiostat ParStat 2273. The electrolytic cell was built by using a 40 mL glass vial with a septum. The working electrode was connected to aluminum foil, while counter and reference electrodes were connected to graphite. The aluminum and the graphite foils were prepared with dimensions of 1 cm × 2.5 cm, exposing an area of 1 cm² in the electrolyte. The applied current was 2 mA. The observed oscillations in the potential could be due to several factors, such as gas bubbling and temperature gradients due to the heating plate.

## Results

**Figure 1:**
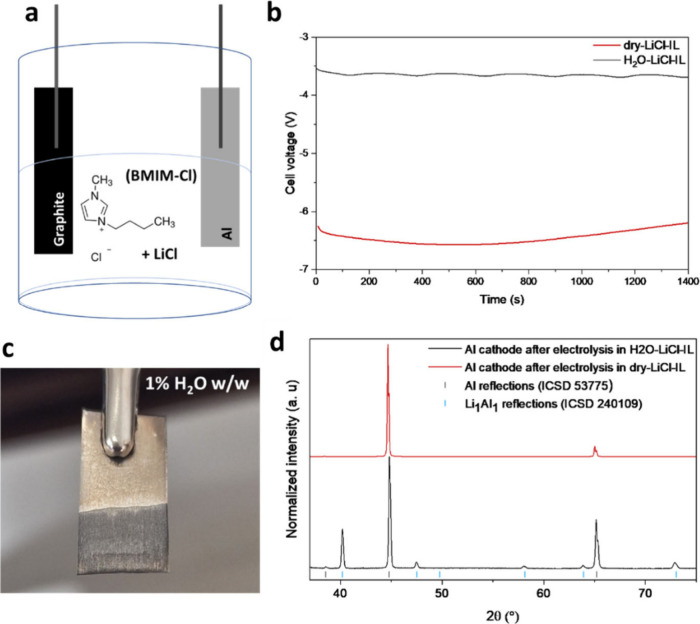
(a) Scheme of the electrolytic cell with Al and graphite electrodes. (b) Galvanostatic profile of electrolysis in H_2_O-LiCl-IL and dry-LiCl-IL electrolytes. (c) Image of the Al foil after electrolysis in the H_2_O-LiCl-IL electrolyte. (d) XRD patterns of the cathode after electrolysis in H_2_O-LiCl-IL and dry-LiCl-IL.

**Figure 2:**
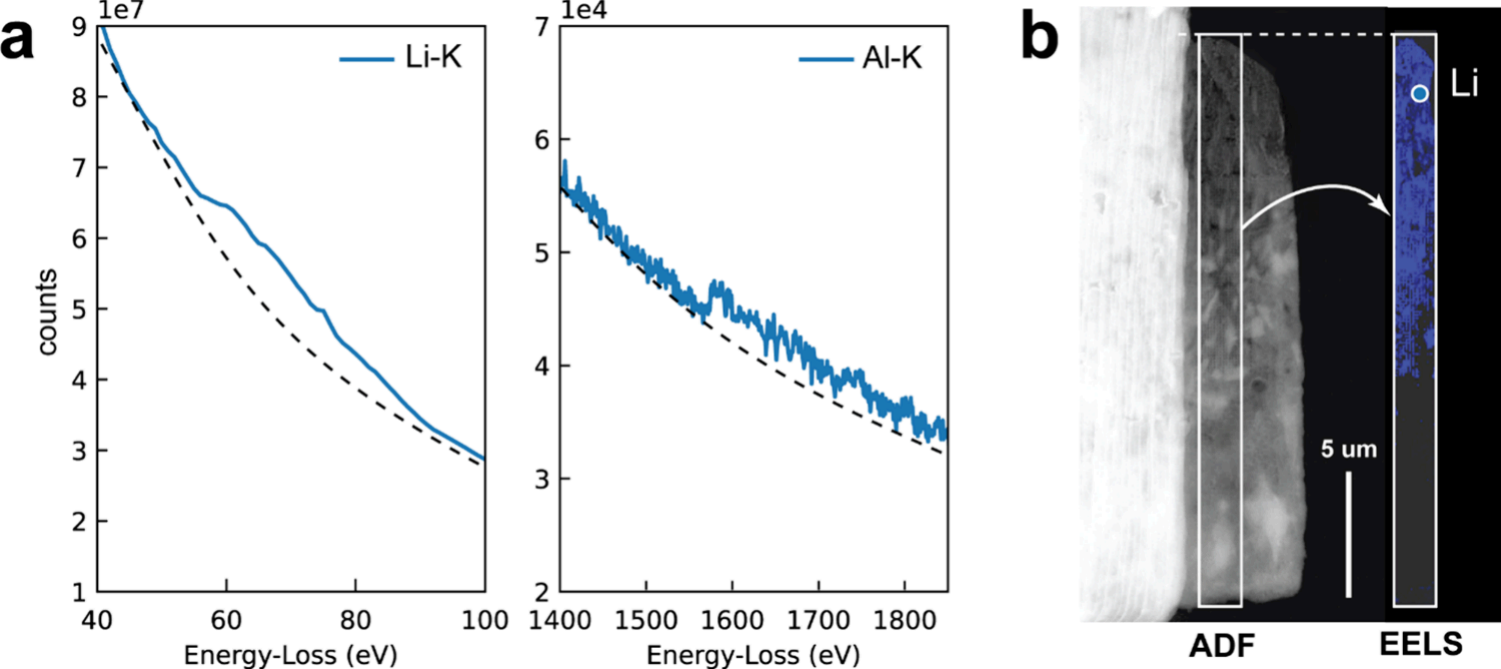
(a) Representative EEL spectrum taken close to the surface and showing the presence of both Al and Li from their respective K ionization edges. (b) Overview (ADF-STEM) of the cross-sectional FIB lamella prepared from the LiAl sample. The sample surface is on top (dashed white line). The distribution of Li as measured from EELS is shown in blue in the map, indicated by the white rectangle. The blue circle marks the region of the sample corresponding to the spectrum shown in panel a.

A LiCl/BMIM-Cl melt containing ca. 1% w/w of water (H_2_O-LiCl-IL) was prepared in a glove box by mixing as-received BMIM-Cl and anhydrous LiCl in a ∼2:1 molar ratio. A vial was directly used as an electrolytic cell with graphite as the positive electrode and a 100-μm-thick Al foil as the negative electrode, as shown in Figure 1a. A second sample (dry-LiCl-IL) was prepared by heating the above LiCl/BMIM-Cl melt under a vacuum to reduce the water content. When a current of 2 mA cm^–2^ was applied between the two electrodes immersed in the H_2_O-LiCl-IL electrolyte at 80 °C, at a potential of ca. −3.5 V (Figure 1b) we observed the evolution of gas bubbles at the graphite electrode while a reaction occurred on the surface of the Al foil leading to the formation of a darker layer (Figure 1c). This layer was supposed (and later confirmed) to be a Li–Al intermetallic compound formed upon reduction of Li^+^ at the surface of Al. By considering the range of potential (−3.5 to −4 V), the possible oxidation reactions occurring at the graphite anode could involve oxide^38−40^ and/or chloride species.^41,42^ On the contrary, when a current of 2 mA cm^–2^ was applied between the two electrodes immersed in the dry-LiCl-IL electrolyte, the potential dropped to ca. −6.5 V (Figure 1b), a value beyond the electrochemical stability window of IL,^43^ and the Al foil was found unchanged upon visual inspection. The electrochemically treated Al foils were characterized by X-ray diffraction (XRD) and gave the patterns shown in Figure 1d. After electrolysis in H_2_O-LiCl-IL, the Al foil displays reflections corresponding to pure Al (ICSD 53775) and to an β-LiAl intermetallic compound (named LiAl in the following; ICSD 240109), demonstrating the successful reduction of Li^+^ at the surface of Al. As expected, the intensity of the LiAl signal is reduced at lower currents (1.0 and 0.5 mA cm^–2^) as shown in Figure S1. On the other hand, only pure Al is detected when dry-LiCl-IL is used as an electrolyte, hinting at the crucial role of water in the process. We suggest that Li^+^ reduction might be hindered in dry-LiCl-IL due to extensive clustering into the polynuclear species [Li_*n*_Cl_*n*+1_]^−^, which might be harder to reduce than the hydrated lithium ions in H_2_O-LiCL-IL. Electrospray ionization mass spectrometry (ESI-MS) indeed showed that signals from [LiCl_2_]^−^, [Li_2_Cl_3_]^−^, and [Li_3_Cl_4_]^−^ species are, on average, less intense in H_2_O-LiCl-IL than in dry-LiCl-IL (see Figure S2 and Tables S1 and S2 in the Supporting Information for more details). The Al foil resulting from the electrolysis in H_2_O-LiCl-IL was also analyzed by electron energy-loss spectroscopy (EELS) in order to measure the depth of the intermetallic region, as shown in Figure 2. To this aim, we used focused-ion beam (FIB) lift-out to prepare a thin cross-section of the sample (lamella) of area 20 μm × 5 μm and ca. 70 nm thickness. We then acquired an EEL spectrum image by scanning a whole portion of the lamella using annular dark field scanning transmission electron microscopy (ADF-STEM) to verify the presence of Li.^44^Figure 2a shows an average spectrum taken from a region close to the surface of the sample in which both Li and Al are detected from the corresponding Li–K and Al–K ionization edges. Figure 2b presents a view of the lamella and the resulting EELS map from the Li–K ionization edge, demonstrating that Li is present within the first ∼10 μm from the surface. Note that electron diffraction revealed that Li exists as Li_2_CO_3_, which likely forms when LiAl reacts with moisture and CO_2_ (see Supporting Information for further details):
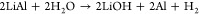

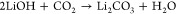
Nevertheless, the distribution of Li provided by EELS is considered to accurately mirror the depth extension of the pristine LiAl phase (Figure S3). We also analyzed the same sample using scanning electron microscopy (SEM): the cross-section image shows an intermetallic thickness of approximately 5–7 μm. The surface appears to exhibit a uniform layer, and it is possible that some residual ionic liquid remains present after the electrolytic process (see Figure S4).

**Figure 3:**
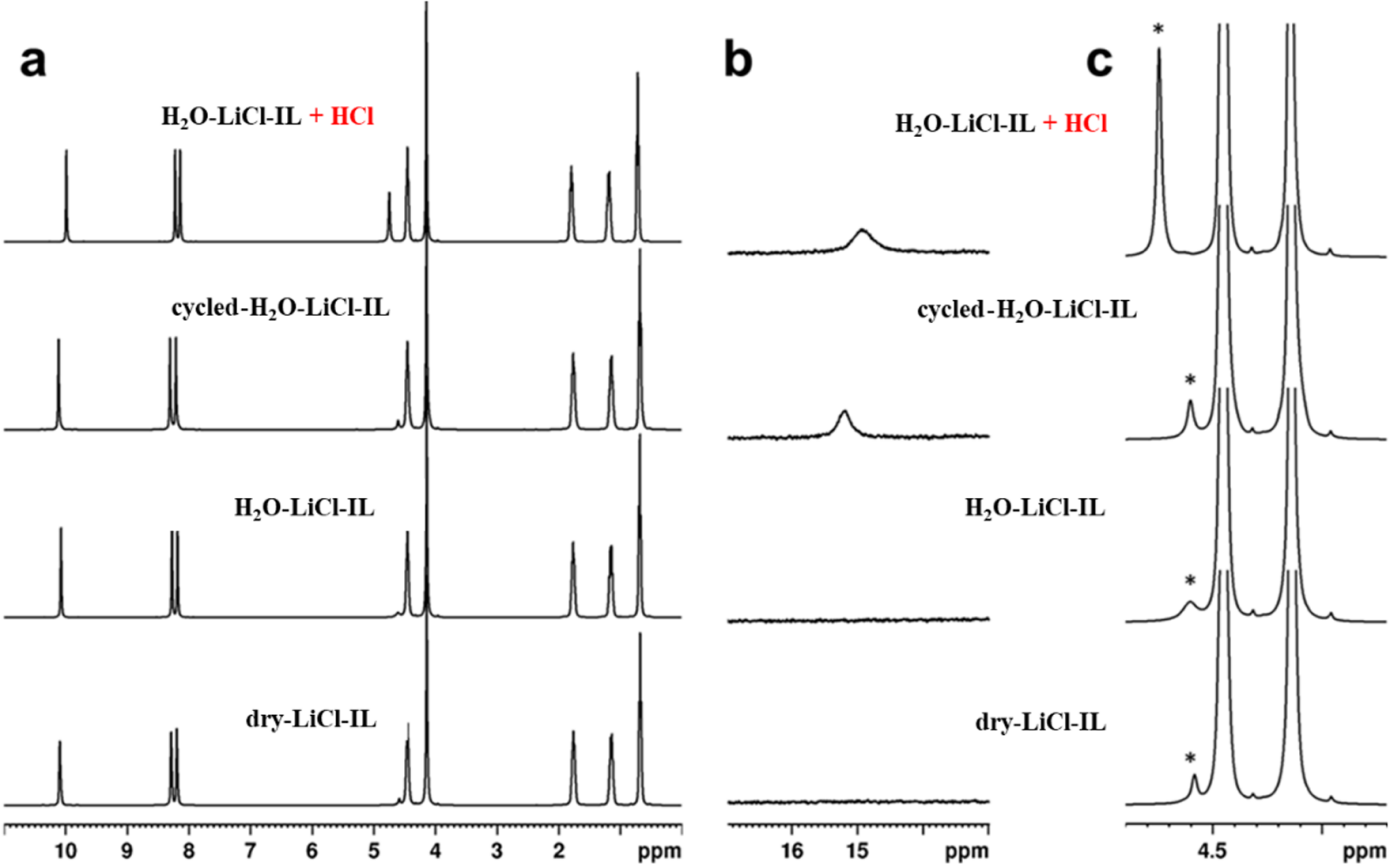
(a) ^1^H NMR spectra at 363 K of (top to bottom) H_2_O-LiCl-IL + HCl, cycled-H_2_O-LiCl-IL, H_2_O-LiCl-IL, and dry-LiCl-IL in the 0–11 ppm region. (b) Enhanced spectral region around 15 ppm showing the signal from acidic protons. (c) Expanded view of the spectral region around 4.3 ppm. The asterisks denote the water signal. The top spectra show the increased intensity of the water signal and the appearance of the broad peak from acidic protons upon the addition of a 1 M aqueous solution of HCl to the H_2_O-LiCl-IL melt. Peak integration gives a water concentration of 4% w/w, which is close to the expected value (3.6% w/w).

Both electrolytes were characterized by NMR along with a third sample, consisting of the H_2_O-LiCl-IL electrolyte after electrolysis, hereafter referred to as cycled-H_2_O-LiCl-IL. The ^1^H NMR spectra at 363 K (Figure 3a) are very similar for the three samples, showing that BMIM-Cl undergoes no significant alteration upon either thermal treatment in a vacuum or electrolysis. We could nevertheless detect the following differences: (i) The peak at 4.6 ppm, attributed to dissolved water, is about twice as intense in H_2_O-LiCl-IL (1.1% w/w) and cycled-H_2_O-LiCl-IL (0.9 %) as in dry-LiCl-IL (0.6 %; Figure 3c). (ii) A weak broad signal typical of acidic protons appears at around 15 ppm after electrolysis (Figure 3b). These assignments were confirmed by the spectral modifications observed upon the addition of aqueous HCl to H_2_O-LiCl-IL (Figure 3, top spectra). The self-diffusion coefficients at 363 K, measured from the decay of BMIM^+ 1^H NMR signals with a sequence based on bipolar gradient pulses, are 5.8 × 10^–12^ m^2^ s^–1^ in dry-LiCl-IL, 6.7 × 10^–12^ m^2^ s^–1^ in H_2_O-LiCl-IL, and 7.6 × 10^–12^ m^2^ s^–1^ in cycled-H_2_O-LiCl-IL. The increase of the self-diffusion coefficient across the series reflects changes in viscosity, which decreases in the order dry- > H_2_O- > cycled-H_2_O-LiCl-IL. The viscosity trend is in agreement with the formation of elemental oxygen rather than chlorine upon electrolysis, as chlorine would lead to an increase in viscosity.^45^

The ^7^Li NMR signal was also acquired at 363 K on dry-LiCl-IL and H_2_O-LiCl-IL. In both samples, the shape of the ^7^Li resonance (Figures S5 and S6) evidences the presence of at least two components, one narrower and one broader. The line widths at half-height, estimated by deconvolution, are 7.4 and 32 Hz in dry-LiCl-IL and 5.4 and 25 Hz in H_2_O-LiCl-IL. The line width of an NMR signal is inversely related to transverse relaxation time (*T*_2_). In turn, *T*_2_ depends inversely on the rotational correlation time (τ_c_), i.e., the average time required by a species to reorient about one radian from its starting position.^46^ The correlation time is a function of temperature (*T*), hydrodynamic volume (*V*), and viscosity (η), as described by the Stokes–Einstein–Debye equation:
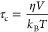


The broader and narrower components can thus be attributed to less and more mobile fractions of Li^+^ ions, respectively. *T*_2_ values were measured from the intensity decays in spin-echo CPMG experiments (Figure S7) fitted with two exponential functions. This gave *T*_2_ = 13.1 and 50.3 ms for dry-LiCl-IL and 16.0 and 52.8 ms for H_2_O-LiCl-IL. Both fractions have shorter *T*_2_’s (i.e. longer Li^+^ correlation times) in dry-LiCl-IL than in H_2_O-LiCl-IL, presumably due to the higher viscosity of the former sample. Unfortunately, we were not able to complement these data with the measured self-diffusion coefficients of Li^+^ ions because the hardware of our NMR spectrometer allowed us to sample only the very beginning of the signal decay. However, after linear interpolation and normalization (Figure S8), we found that the signal decay, although very slow in both cases, is slightly faster in H_2_O-LiCl-IL than in dry-LiCl-IL, corresponding to a faster diffusion of Li^+^ ions in the former sample.

**Figure 4:**
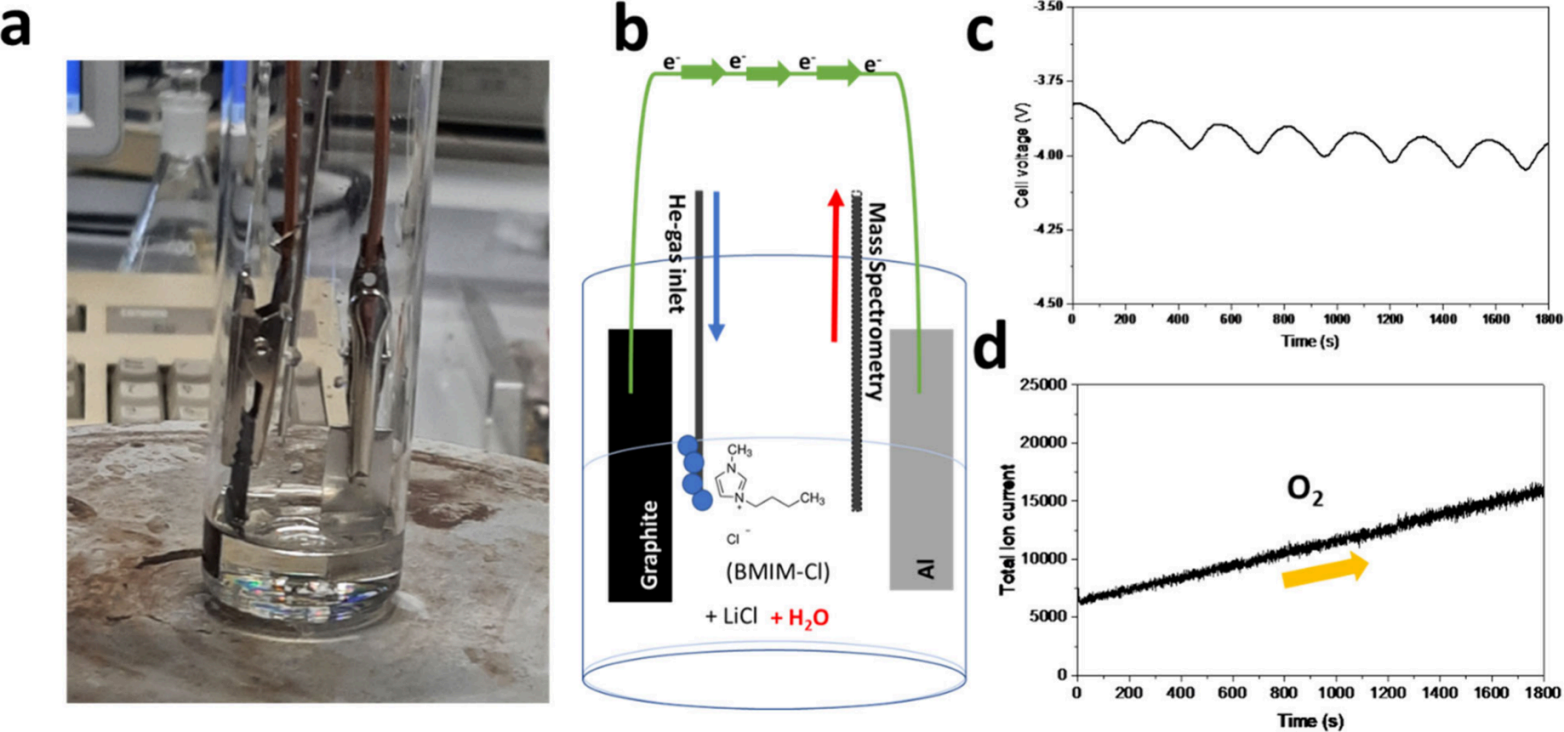
(a,b) Electrolytic cell equipped for in situ gas analysis by MS. (c) Voltage profile during the charging process. (d) O_2_ signal during the charging process (*m*/*z* = 32).

The processes inside the electrolyte-loaded cell were also probed by mass spectrometry (MS) with the dual objective of (1) monitoring gas evolution in order to unveil the reaction pattern at the graphite anode and (2) analyzing any compositional differences before and after electrolysis by means of a solid phase microextraction (SPME) device combined with a gas chromatographic system interfaced to a quadrupole spectrometer (SPME-GC/MSq). The evolution of gas during the electrolysis process was determined by means of the instrumental setup shown in Figure 4a,b. In operando gas detection was carried out using a mass spectrometer directly connected to the electrolytic cell. A gas inlet capillary was dipped into the electrolyte close to the graphite anode, and He was bubbled to extract any gas formed during the charging process reported in Figure 4c (see section S.2.6 in the Supporting Information for more details). A gas outlet capillary was positioned near the surface of the electrolyte and directly connected to the mass analyzer. The O_2_ signal (*m*/*z* = 32) was found to increase linearly with time as electrolysis proceeded (Figure 4d), in agreement with Freiderich^47^ Note that contamination by O_2_ from air can be excluded since the signal of N_2_ (*m*/*z* = 28) remained constant during the analysis.

In addition to forming a LiAl intermetallic at the cathode and producing oxygen at the anode, the electrolytic process also alters the composition of the headspace inside the cell. In fact, the gas chromatographic traces obtained from the thermal desorption of the SPME device before the start of the electrolysis (Figure S9) and during the electrolysis (Figure S10) show some differences, with the disappearance of some signals (analytes) and the appearance of signals corresponding to species with a lower molecular weight. Table S3 reports the main chemical species identified in the headspace of the electrolytic cell. Specifically, the analysis of the chromatographic traces immediately highlights, on one hand, the compositional complexity of the gas phase in equilibrium with the solution and, on the other hand, its variation during the electrolytic process. In particular, we observed the disappearance of 1-methylimidazole and 1,4-dimethylimidazole which were present inside the cell before the start of the electrolysis. We detected the formation of gaseous molecules like 4,5-dichloro-1-methylimidazole.

**Figure 5:**
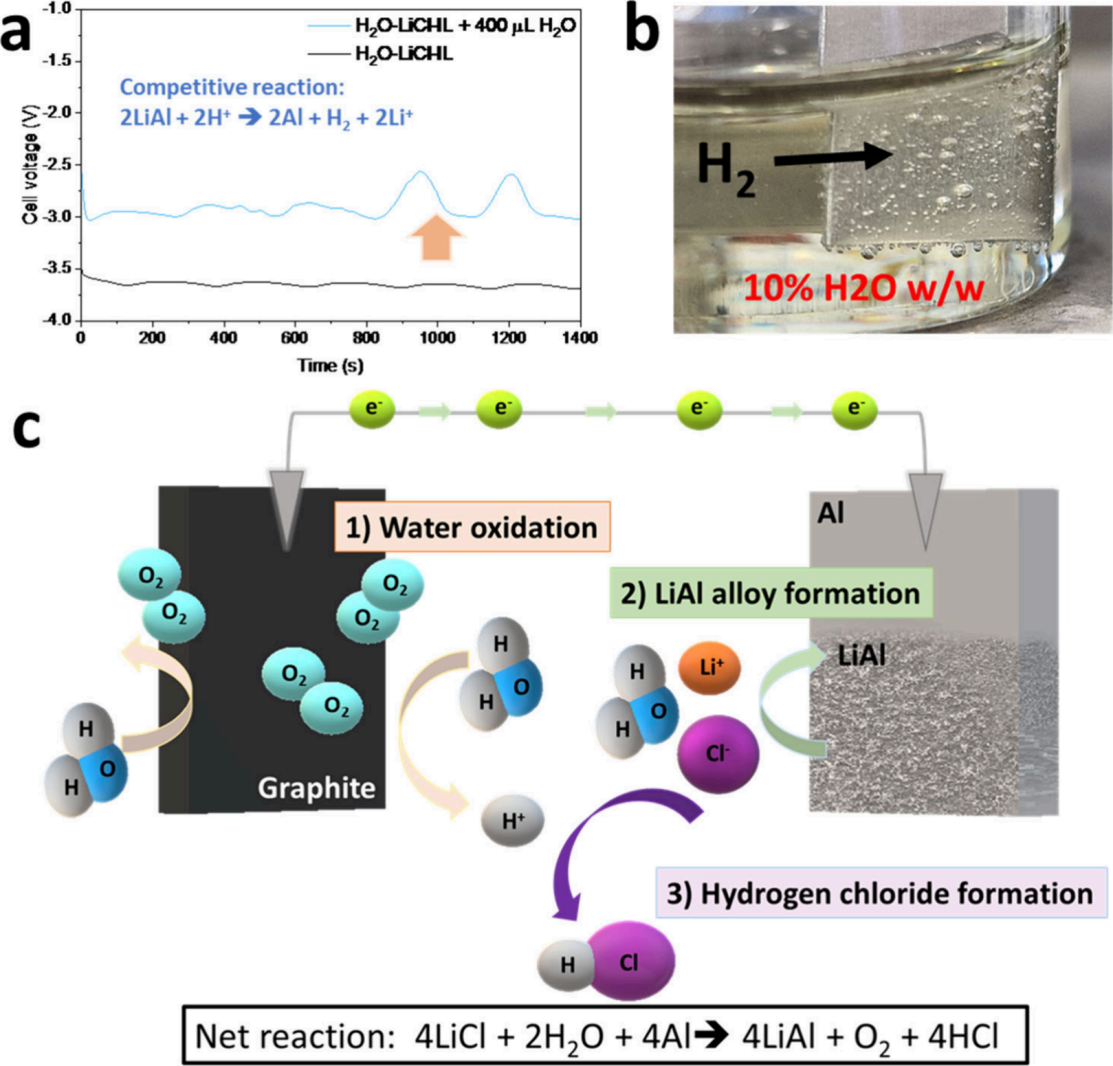
(a) Galvanostatic profile of H_2_O-LiCl-IL before and after the addition of 400 μL of H_2_O to increase the water concentration from ca. 1% to ca. 10% w/w. (b) H_2_ evolution at the Al surface during the electrolysis of H_2_O-LiCl-IL containing ca. 10% w/w of H_2_O. (c) Reaction mechanism to form LiAl intermetallic, oxygen gas, and hydrogen chloride.

As shown in Figure 5a, when the electrolysis was performed with a higher water concentration (ca. 10% w/w), the operating voltage increased to −3.0/–2.5 V. This potential is consistent with the formation of H_2_ (Figure 5b) instead of LiAl intermetallic at the cathode. We also explored intermediate amounts of water (ca. 3, 5, and 8% w/w). During the electrolysis of samples containing 3% and 5% water, a slight decrease in potential (approximately 0.1 V) was observed during the process (Figure S11a). However, up to 5% water, the formation of the LiAl intermetallic is still possible, although the XRD patterns in Figure S11b indicate that the amount of LiAl decreases significantly passing from 1% to 5% water. Regarding the sample with an 8% water content, the operating voltage during the electrolysis was found to be intermediate between those recorded with 1% and 10% water, and no LiAl intermetallic is detected by XRD (as found for the 10% sample). Therefore, a water percentage of 1% w/w is optimal to promote LiAl electrodeposition over the evolution of H_2_. We evaluated the efficiency of the electrolysis process for this 1% sample, obtaining a value of 98.2% (see Figure S12 for more details).

Our findings support the mechanism reported in Figure 5c: (a) The formation of LiAl intermetallic is possible only if water is present. (b) During charging, O_2_ is evolved (as detected by MS) and HCl is formed into the melt (as detected by NMR analysis). (c) When the amount of water is ca. 1% w/w, the deposition of LiAl is highly preferential. (d) With increasing water content, both LiAl and H_2_ are formed at the cathode until, at water concentrations of 8% w/w (or above), water reduction to H_2_ overrides the formation of LiAl. The absence of Cl_2_ in the melt is also confirmed by the analysis of gases evolved at room temperature and during heating to 200 °C (see section S.2.7 in the Supporting Information). Figure S13 shows that only O_2_ is released from the melt at room temperature. Once O_2_ is removed through a controlled He flow and the sample is heated to 200 °C, the release of both O_2_ (which also occurs at room temperature) and HCl, but not Cl_2_, can be observed. We suppose that in our system the evolution of Cl_2_ at the anode, which would lead to an operating voltage of ca. −3.3 V, is kinetically disfavored over water oxidation to O_2_. The whole process can be summarized as follows:
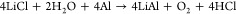
while the possible competitive reaction is
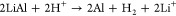


Our process offers interesting perspectives for LiAl production: water acts as a sacrificial agent during the charging process, forming O_2_ which can be collected. The dissolved HCl can be neutralized by adding a strong base in order to restore the original pH value. The addition of fresh LiCl and water (1% w/w) can be effective enough to continue the reduction process in a second step.

## Conclusions

In conclusion, we demonstrated a novel process to produce LiAl intermetallic films, in which water plays a crucial role as a sacrificial agent. Electrolysis of a solution of LiCl in an ionic liquid containing ca. 1% w/w of water yields LiAl at the cathode and O_2_ (but not Cl_2_) at the anode with concomitant formation of HCl as a byproduct. In this way, we were able to fabricate micrometer-thick LiAl films at low temperatures, offering a simple and efficient approach. The intermetallic films were characterized by complementary techniques such as XRD and EELS while the ionic liquid was analyzed by MS and NMR. These findings are promising for the development of LiAl-based materials with potential applications in energy storage and materials science.

## Supporting Information Available

Experimental details about XRD, NMR, SEM, TEM, EELS, and GC-MS (PDF)
