# Supplementary material for: Water-Assisted Electrosynthesis of a Lithium–Aluminum Intermetallic from a Lithium Chloride-Ionic Liquid Melt
Source: ACS Electrochem. 2025 Jan 17;1(5):599–606. doi: 10.1021/acselectrochem.4c00134 (PMC12051454; doi:10.1021/acselectrochem.4c00134)
Supplement: Supplementary file 1 — ec4c00134_si_001.pdf [file ec4c00134_si_001.pdf]

SUPPORTING INFORMATION

Water-assisted Electrosynthesis of a Lithium-Aluminum Intermetallic from a Lithium Chloride-Ionic Liquid Melt

Fabrizio Bernini[1,†], Giovanni Bertoni[2,†], Adele Mucci[1,†], Andrea Marchetti[1], Daniele Malferrari[1], Gian Carlo Gazzadi[2], Marco Ricci[3], Sergio Marras[3], Remo Proietti Zaccaria[3], Enzo Rotunno[2], Alessio Nicolini[1], Nassima Yamini[1], Andrea Cornia[1], Marco Borsari[1], and Andrea Paolella[1,3,*]

[1] Dipartimento di Scienze Chimiche e Geologiche e UdR INSTM, Università degli Studi di Modena e Reggio Emilia, Via G. Campi 103, 41125 Modena, Italy

[2] CNR – Istituto Nanoscienze, Via G. Campi 213/A, 41125 Modena, Italy

[3] Istituto Italiano di Tecnologia, Via Morego 30, 16163 Genova, Italy

[†] These authors equally contributed to the manuscript

*Corresponding author: Prof. Andrea Paolella andrea.paolella@unimore.it

Contents

Materials and Methods p. S2

Figure S1. XRD patterns of the cathode after electrolysis in **H_2O-LiCl-IL** at different currents p. S5

Figure S2. ESI-MS spectra of **dry-LiCl-IL** and **H_2O-LiCl-IL** p. S6

Table S1. ESI-MS results in positive ion mode p. S6

Table S2. ESI-MS results in negative ion mode p. S6

Figure S3. Evidence of Li_2CO_3 formation after exposure to air p. S7

Figure S4. Cross section SEM image of **H_2O-LiCl-IL** after electrolysis p. S7

Figure S5. ^{7}Li NMR signals of **dry-LiCl-IL** and **H_2O-LiCl-IL** p. S8

Figure S6. Deconvolution of ^{7}Li NMR signals of **dry-LiCl-IL** and **H_2O-LiCl-IL** p. S8

Figure S7. ^{7}Li NMR signal decay as a function of the echo time in CPMG experiments p. S9

Figure S8. ^{7}Li NMR signal decay in diffusion measurements p. S9

Figure S9. In situ GC profile before starting the electrolysis process p. S10

Figure S10. In situ GC profile during the electrolysis process p. S10

Figure S11. Galvanostatic profiles and XRD patterns of the Al cathode after electrolysis in electrolytes containing variable amounts of water p. S11

Figure S12. Galvanostatic profile of **H_2O-LiCl-IL** over 6.5 h p. S11

Figure S13. Mass analysis of gases released from cycled **H_2O-LiCl-IL** p. S12

Table S3. Retention time, name, experimental conditions (no electrolysis/electrolysis) and % match for chemical species detected by SPME-GC/MS technique p. S13

Materials and methods

S.1 Materials

BMIM-Cl (99.9%) and anhydrous lithium chloride (99.98% metal basis) were purchased from Iolitec (Germany) and Sigma Aldrich, respectively. A 100 μm aluminum foil and a graphite foil were purchased from Sigma Aldrich.

S.2 Experimental methods

S.2.1 X-ray diffraction (XRD)

XRD patterns were recorded on a Malvern-PANalytical 3rd generation Empyrean X-ray diffractometer equipped with a 1.8 kW CuKα ceramic X-ray tube operating at 45 kV and 40 mA, automated prefix iCore-dCore optical modules for the incident and diffracted beam paths, PIXcel3D 2x2 area detector. The diffraction patterns were collected in Bragg-Brentano geometry using a reflection-transmission spinning sample stage (rotation speed = 2 rps). Air-stable samples were sealed between two layers of 6 μm-thick Mylar® foil, while air-sensitive samples were sealed between two layers of 7 μm-thick Kapton® foil lined with vacuum grease.

S.2.2 Electrospray ionization mass spectrometry (ESI-MS)

ESI-MS measurements were conducted in both positive and negative ion modes on a 6310A Ion Trap LC-MS(n) instrument (Agilent Technologies) by direct infusion of CH_2Cl_2/*i*PrOH solutions (~10:1 v/v). In this solvent mixture, both **dry-LiCl-IL** and **H_2O-LiCl-IL** are completely soluble. CH_2Cl_2 was purchased anhydrous and stored over activated 4Å molecular sieves. *i*PrOH was stored over activated 3Å molecular sieves prior to use.

S.2.3 Nuclear magnetic resonance (NMR)

The samples were melted at a temperature of 100 °C and transferred into an NMR tube using a Pasteur pipette. NMR spectra were obtained with a Bruker NMR Avance Neo 400 MHz spectrometer, operating at 400.13 MHz for ^{1}H and at 155.51 MHz for ^{7}Li. The ^{1}H NMR spectra were obtained at 363 K with a 5 mm probe for liquids and a standard pulse-and-acquire sequence, without lock on deuterium. The ^{7}Li NMR spectra were also obtained at the same temperature on the same samples used for ^{1}H NMR measurements. Diffusion measurements were done with a sequence based on stimulated echo and bipolar gradient (ledbpgp2s, Bruker library), with big delta (Δ) 100 ms (^{1}H) or 300 ms (^{7}Li), eddy current delay 5 ms, little delta (δ) 2 × 2.5 ms, 32 points linear ramp (from 1 to 47 G/cm) of sine-shaped gradients. The self-diffusion coefficients were derived using Dynamics Centers 2.4.10 software (Bruker) and single-exponential decays, fitting the experimental data with the equation:

$$I = I_0\, e^{-D\gamma^2 g^2 \delta^2 (\Delta - \frac{\delta}{3})}$$

where D is the apparent self-diffusion coefficient, γ the nucleus gyromagnetic ratio, and g the gradient strength. ^{7}Li diffusion measurements in **dry-LiCl-IL** required a number of scans twice as large as that used for **H_2O-LiCl-IL** in order

to compensate for the faster decay of ^{7}Li signal during the long diffusion time. A Carr-Purcell-Meiboom-Gill (CPMG) spin-echo sequence (cpmg, Bruker library) was used to measure ^{7}Li transverse relaxation time (T_2) with ten time points in the range 5-350 ms. The signal decays were interpolated with Dynamics Centers 2.4.10 software (Bruker) using a sum of two-exponential functions:

$$I = I_0 e^{-\frac{t}{T_{2_0}}} + I_1 e^{-\frac{t}{T_{2_1}}}.$$

The linewidths at half-height were estimated by deconvolution using MestReNova 15.0.1 (Mestrelab Research S.L.).

S.2.4 Transmission electron microscopy (TEM)

Transmission electron microscopy (TEM) images taken in scanning mode (ADF-STEM) and spatial resolved electron energy-loss spectroscopy (EELS) were performed on a ThermoFisher Scientific Talos 200S microscope, operated at 200 kV and equipped with a post-column spectrometer (Gatan Inc.) to measure the Li-K and Al-K ionization edges. The Li-K edge was quantified using a Hartree-Slater cross section. The distribution of Li was monitored in EELS in a cross-sectional view of an ultrathin lamella prepared by focused-ion beam (FIB) milling, performed on a FEI Strata DB235M. A slab, obtained by high current milling (5 nA, 30 keV), was extracted from the sample using an *in situ* nanomanipulator (lift-out procedure) and attached to a TEM grid where it was thinned down by low current milling (100 pA, 30 keV) followed by a final polishing at lower ion energy (5 keV). After exposure to air and the electron beam, we noted the formation of a Li_2CO_3 phase (zabuyelite) as revealed by electron diffraction (ED).

S.2.5 Scanning electron microscopy (SEM)

SEM imaging was conducted using a FEI Helios Nanolab 650 DualBeam scanning electron microscope-focused ion beam (SEM-FIB) system. The micrographs were acquired at an accelerating potential difference of 5 kV. The cross-section was obtained by employing the included Tomahawk™ ion column by accelerating gallium ions with a potential difference of 30 kV.

S.2.6 Mass spectrometry (MS)

The vial containing the solution to be electrolyzed and the two electrodes was modified to include a system of in- and out- capillaries to allow a flow of He gas. One of the two capillaries, the gas-in one, was immersed in the solution near the graphite in order to favor the stripping of gas through the electrolytic solution. The gas-out capillary, left in the head space of the vial, was connected directly to the transfer line of the spectrometer in order to send the gas flow coming from the cell directly into the electron impact (EI) ionization interface operating at 70 eV and in positive ion mode. The flow of He inside the vial (approximately 1 mL min^{-1}) was regulated with a flowmeter valve by monitoring the degree of vacuum inside the spectrometer with the aim to not to alter excessively the high vacuum conditions of the mass spectrometer. Before starting the measurement process, the system was conditioned by He gas flow for about 30 minutes to remove any traces of oxygen. Subsequently, the electrolysis cycle was started together with the monitoring

of the total ion current parameter of the spectrometer in the *m/z* range from 10 to 50 amu in mass scan mode. During the electrolytic process, a current of 2 mA cm^{-2} was applied for 30 minutes. To visualize the presence of oxygen and verify the trend during the electrolytic process it was necessary to extract the current contribution of the ion at *m/z* = 32 from the TIC chromatogram vs. time, as shown in **Figure 4c-d**.

The recognition of the compounds sampled with SPME technique was achieved by comparing the experimental mass spectra with NIST05 library ones. The structure assignment is considered positive with a probability index greater than 85%.

S.2.7 Evolved gas mass analysis

To confirm the absence of Cl_2 in the **cycled-H_2O-LiCl-IL** , analysis of gases emitted at room temperature and during heating to 200 °C was performed. Approximately 4 g of the **cycled-H_2O-LiCl-IL**were placed in a sealed vial equipped with a capillary that allowed He to enter at a flow rate of 100 mL min^{-1}, a vent valve, and the inlet-capillary of a gas mass spectrometer (ESS GeneSys Quadstar 422, ESS Ltd, Cheshire CW9 7LU, UK). Mass analyses were performed in multiple ion detection mode by measuring the *m/z* ratios 16 and 32 for O_2, 35, 36, and 37 for HCl, 70 and 72 for Cl_2. A SEM (Secondary Electron Multiplier) detector set at 900 V with an integration time of one second for each *m/z* measured was used to collect the signals. Heating was done by partially dipping the vial in an oil bath placed on a heating stirrer. The results of the experiment are shown in **Figure S13**. At zero time, at room temperature and keeping the vent valve open, He flow was initiated, which purged the atmosphere inside the vial; indeed, a progressive decrease in O_2 signals (m/z = 16, 32) occurred. After 20 min, the flow of He was stopped and the vent valve closed. After about 1 min, a gradual increase in O_2 signals by release from H_2O-LiCl-IL was observed, as O_2 is also released at room temperature. As soon as the O_2 signal was stabilized (about 30 min from the start of the experiment), the vial was dipped into the oil bath at 200 °C, and once H_2O-LiCl-IL was molten, it was gently stirred. After about 2 minutes, a gradual increase in HCl signals (m/z = 35, 36, 37) occurred, indicating its release from the molten material; the significant decrease in O_2-related signals is due to a reduction in O_2 partial pressure. The HCl signals increase up to about 45 min and then decrease due to the gradual depletion of HCl in the molten material.

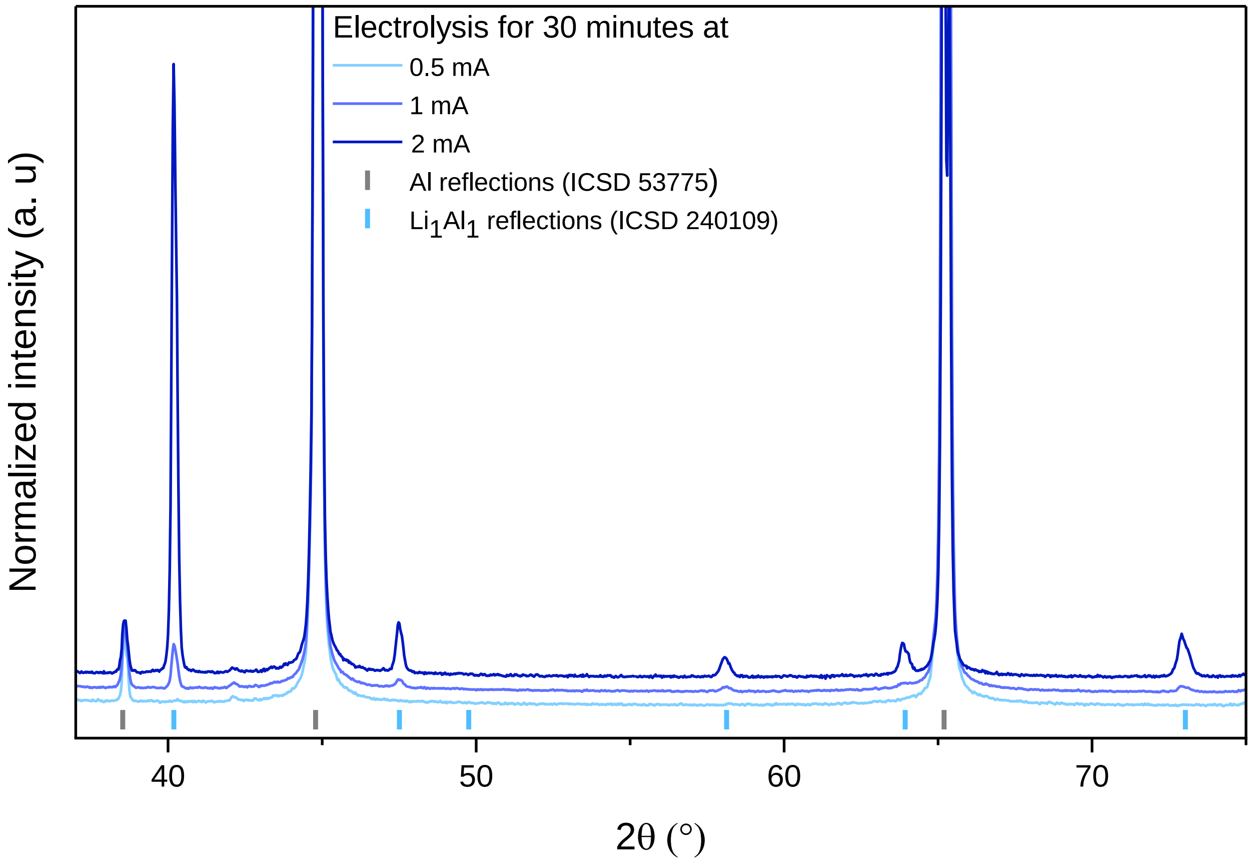

Figure S1: *XRD patterns of Al foil samples after electrosynthesis in H_2O-LiCl-IL for 30 minutes at 0.5 (light blue), 1 (indigo), and 2 (blue) mA cm^{-2}.*

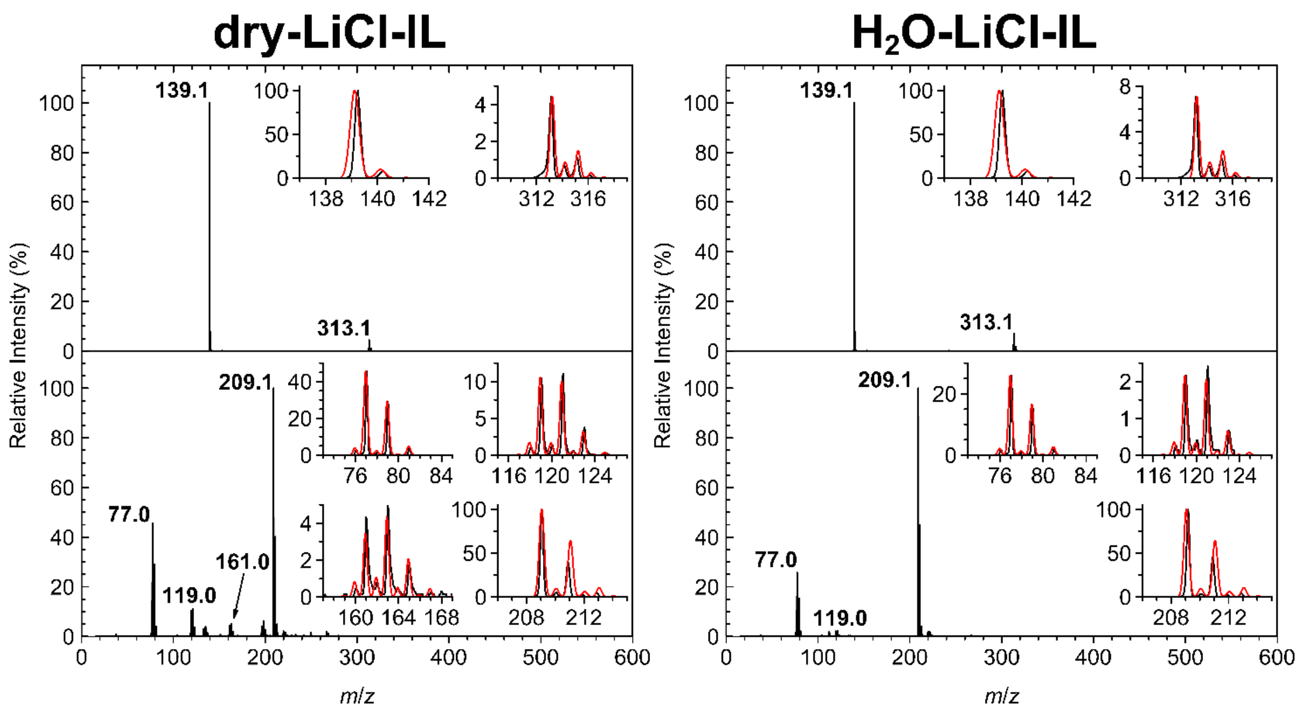

Figure S2. *ESI-MS spectra of dry-LiCl-IL and H_2O-LiCl-IL (CH_2Cl_2/iPrOH ~10:1 v/v) in positive (upper panels) and negative (lower panels) ion modes. The insets show the experimental (black line) and simulated (red line) isotopic patterns of the species.*

Table S1. *ESI-MS results in positive ion mode.*

		Relative intensity (%)	
m/z	**Species**	**dry-LiCl-IL**	**H_2O-LiCl-IL**
139.1	$[BMIM]^+$	100	100
313.1	$[(BMIM)_2Cl]^+$	4	7

Table S2. *ESI-MS results in negative ion mode.*

		Relative Intensity (%)	
m/z	**Species**	**dry-LiCl-IL**	**H_2O-LiCl-IL**
77.0	$[LiCl_2]^-$	46	26
119.0	$[Li_2Cl_3]^-$	11	2
133.0	?	3	traces
161.0	$[Li_3Cl_4]^-$	4	traces
195.9	?	4	traces
209.1	$[(BMIM)Cl_2]^-$	100	100

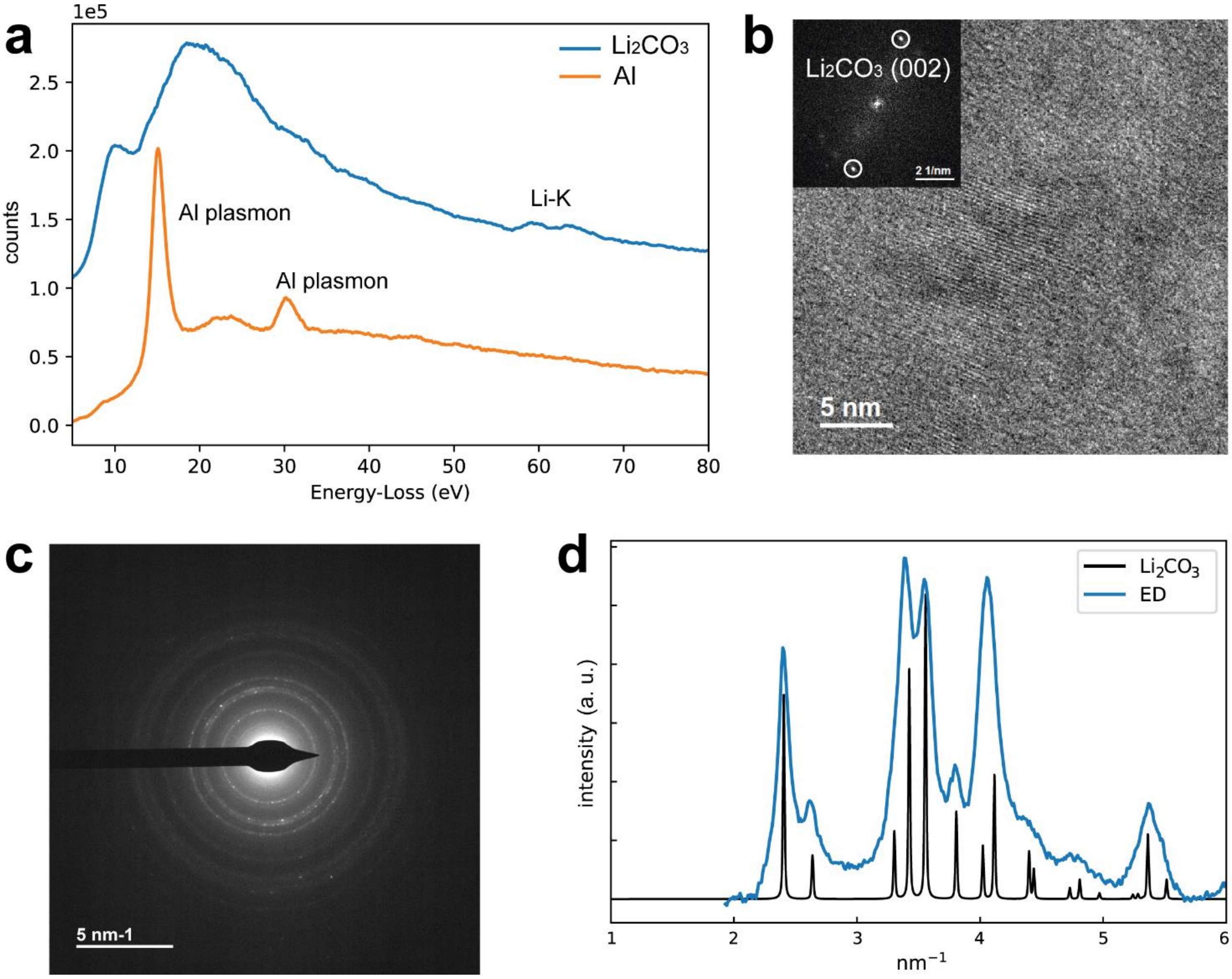

Figure S3. *Evidence of Li_2CO_3 formation after exposure to air. a) Two representative low-loss spectra taken from a second acquisition across the FIB lamella. Deep in the sample (ca. 15 μm below the surface) a typical spectrum from metallic aluminum (orange curve) is recognized from its plasmon peaks at 15 eV and 30 eV. Close to the surface, a spectrum containing Li (blue curve) is probably to be addressed as Li_2CO_3. b) HRTEM image taken close to the surface, confirming the presence of Li_2CO_3 from its (002) planes at 0.28 nm (FFT in the inset). c) Electron diffraction (ED) from a region close to surface and d) its azimuthal integration, which fits well with the reflections expected from polycrystalline Li_2CO_3 (Zabuyelite). These evidences confirm the reaction of Li from the LiAl alloy after exposure to air.*

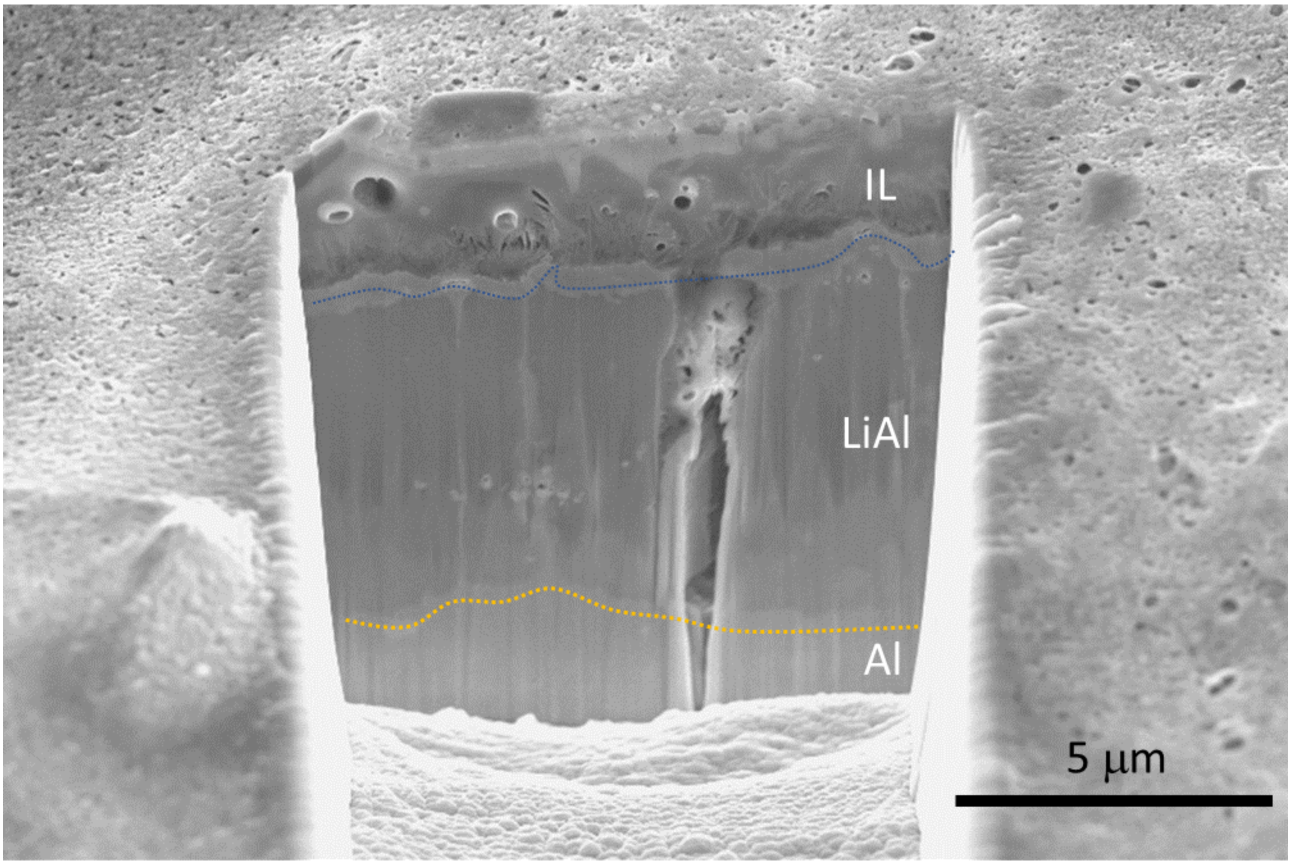

Figure S4. *Cross-sectional scanning electron micrograph of Al foil after the electrosynthesis process in H_2O-LiCl-IL.*

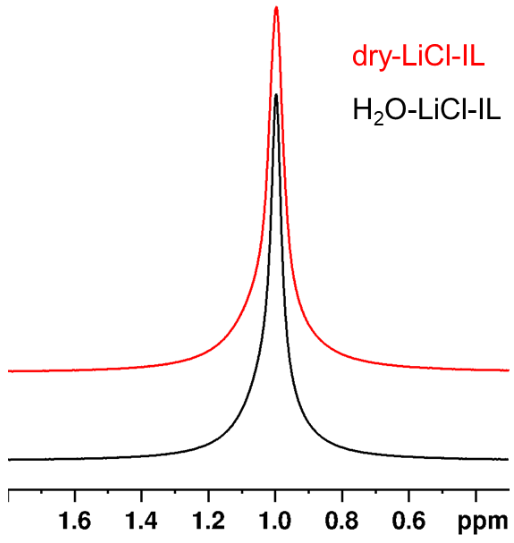

Figure S5. *^{7}Li NMR signals of dry-LiCl-IL (red) and H_2O-LiCl-IL (black).*

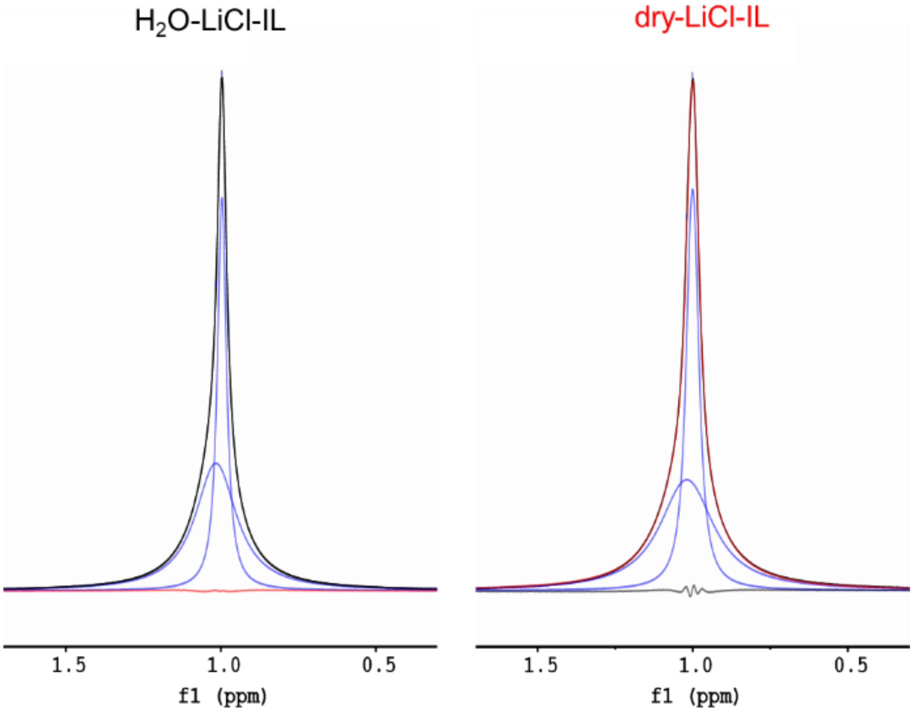

Figure S6. *Deconvolution of ^{7}Li NMR signals of dry-LiCl-IL (red) and H_2O-LiCl-IL (black).*

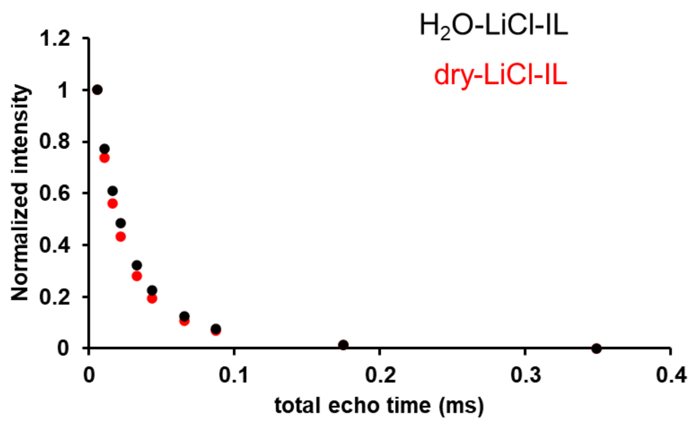

Figure S7. *^{7}Li NMR signal decay as a function of the echo time in CPMG experiments.*

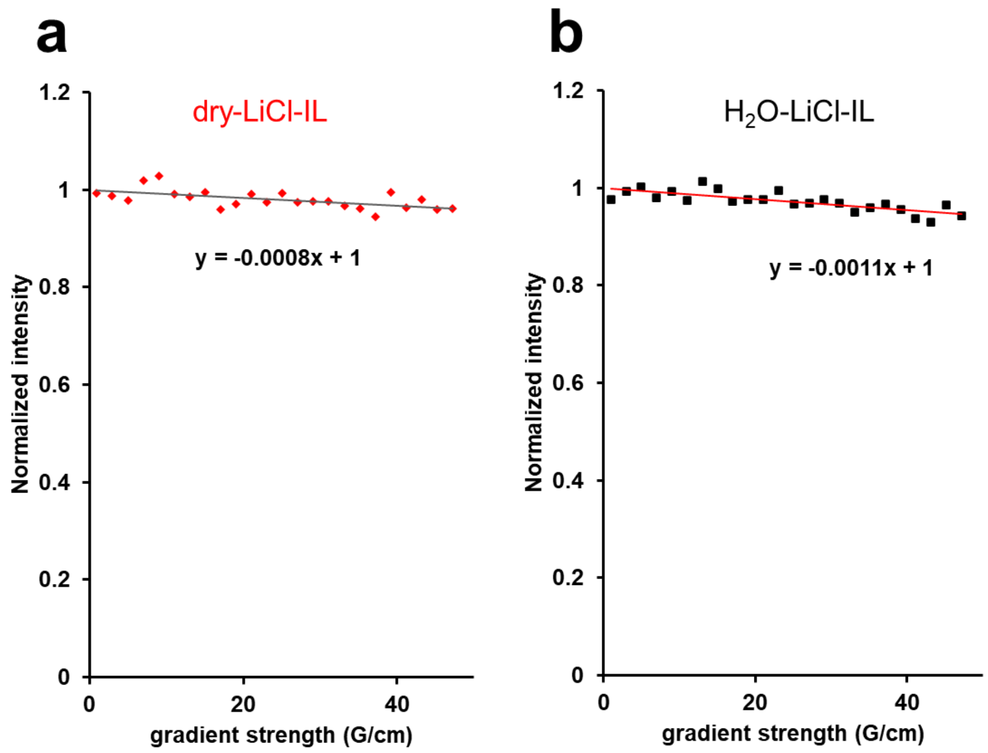

Figure S8. *^{7}Li NMR signal decay in diffusion measurements. The initial signal decay was approximated to a straight line.*

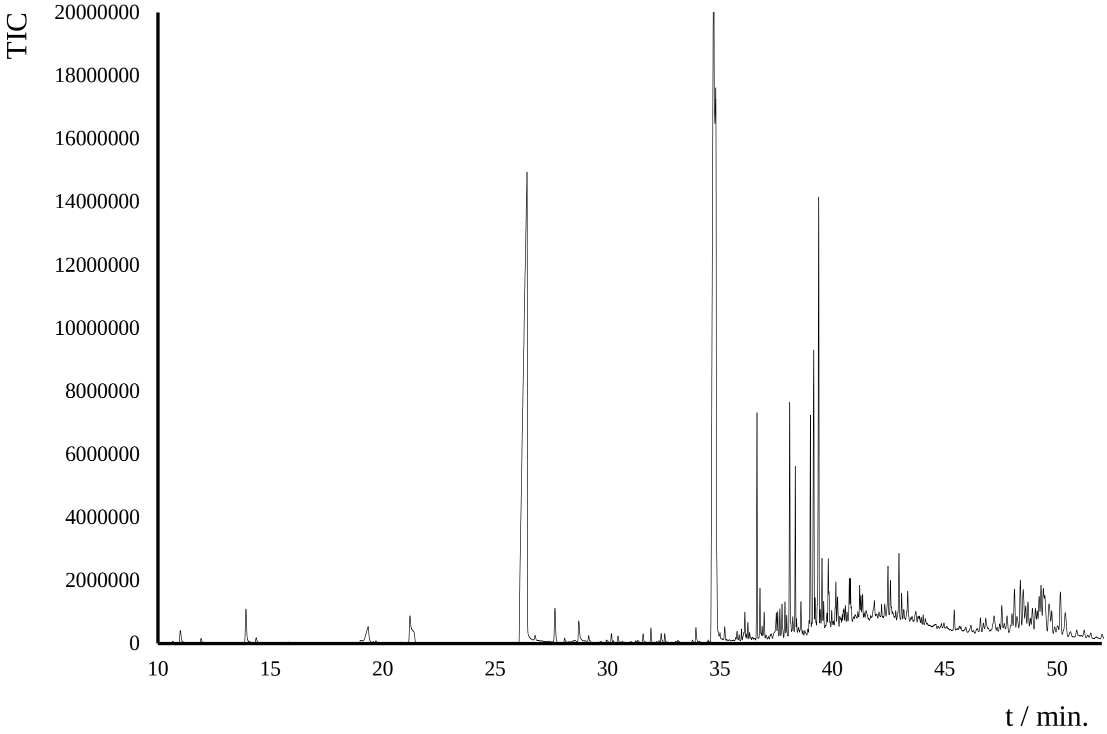

Figure S9. *In situ GC profile before starting the electrolysis process.*

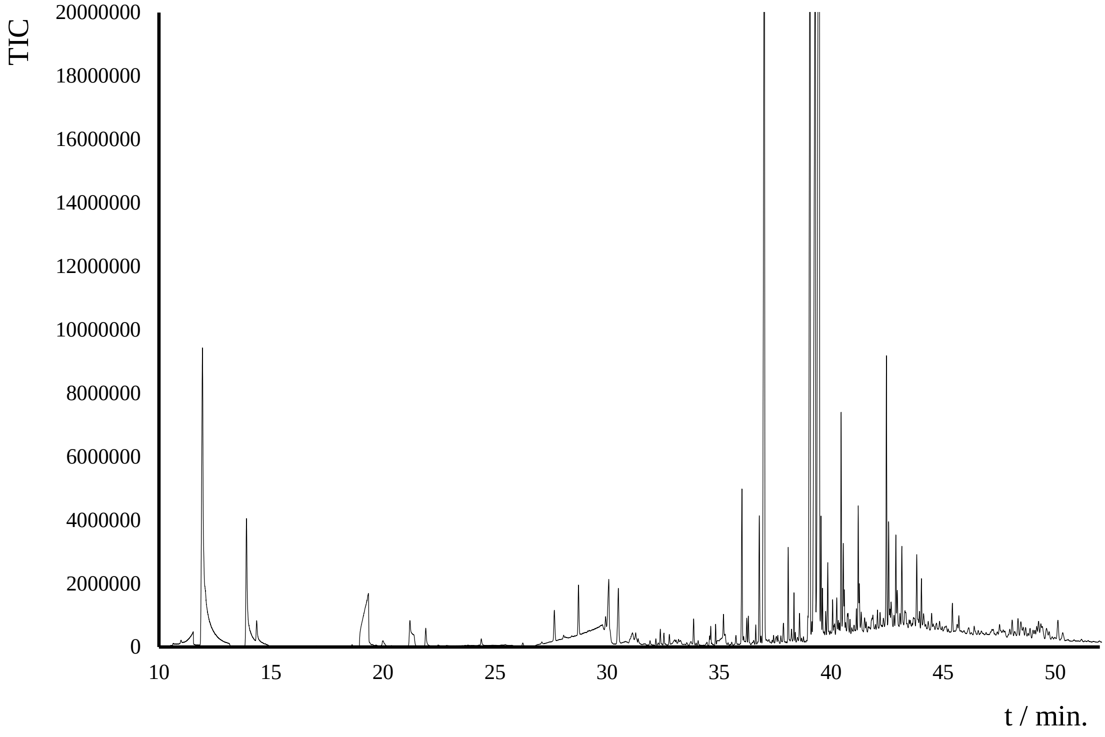

Figure S10. *In situ GC profile during the electrolysis process.*

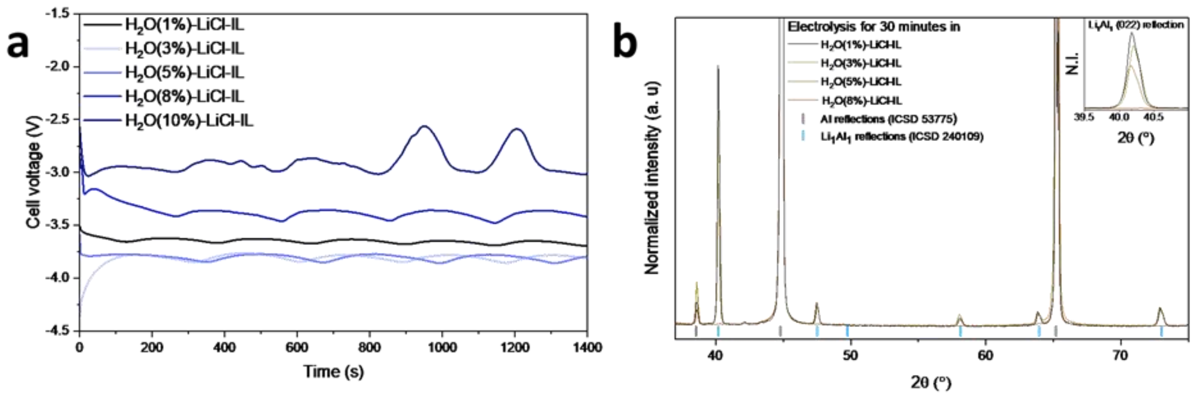

Figure S11. *a) Cell potential profile during the electrosynthesis process in H_2O-LiCl-IL containing 1% (black), 3%, 5%, 8%, and 10% (progressively darker blue tone) H_2O. b) XRD patterns of Al electrodes after electrosynthesis for 30 minutes in H_2O(1%)-LiCl-IL (black), H_2O(3%)-LiCl-IL, H_2O(5%)-LiCl-IL, and H_2O(8%)-LiCl-IL (progressively darker yellow tones).*

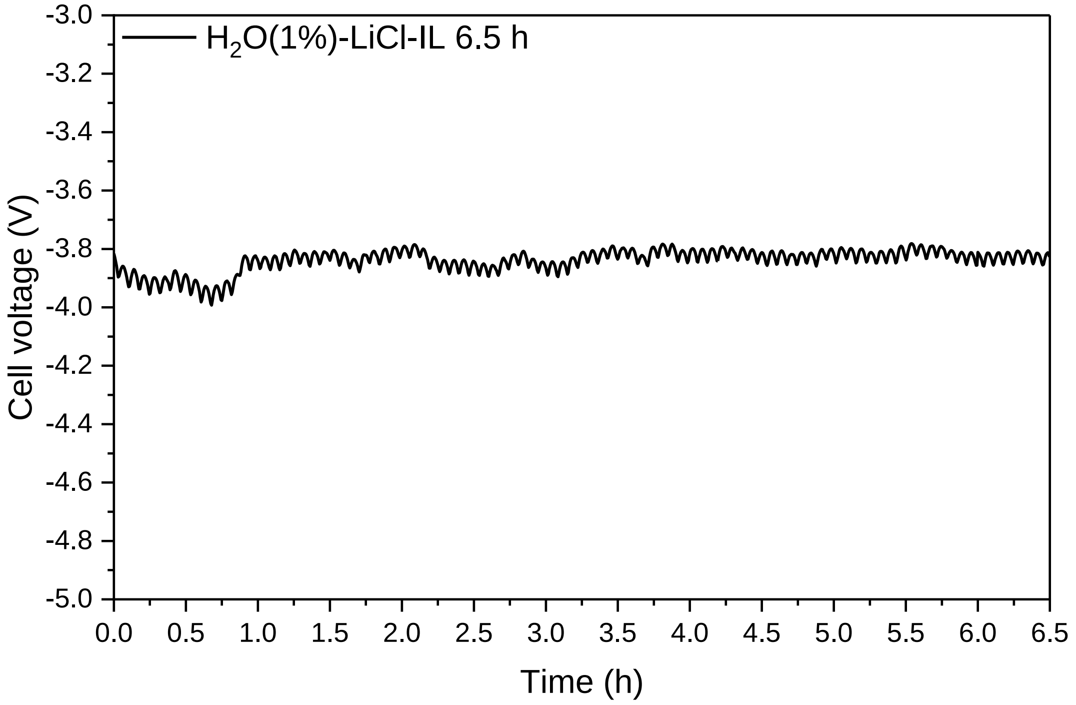

Figure S12. *Cell potential profile during the electrosynthesis process in H_2O(1%)-LiCl-IL sample for 6.5 h.*

The theoretical mass of reduced lithium (m_{Li}) can be calculated using the formula:

$$m_{\mathrm{Li}} = \frac{M_{\mathrm{Li}} \cdot Q}{F \cdot n}$$

where M_{Li} is the molar mass of lithium (6.94 g mol^{-1}), Q is the total charge passed (in C), F is Faraday's constant (96485 C mol^{-1}), and $n = 1$ is the number of electrons involved in the reduction reaction. Using a current of 2 mA for 6.5 hours, the total charge Q passed is 46.8 C (0.013mAh * 3600). We find $m_{\mathrm{Li}} = 3.36$ mg, to be compared with an experimental mass of reduced lithium of 3.30 mg, resulting in a process efficiency of 98.2%.

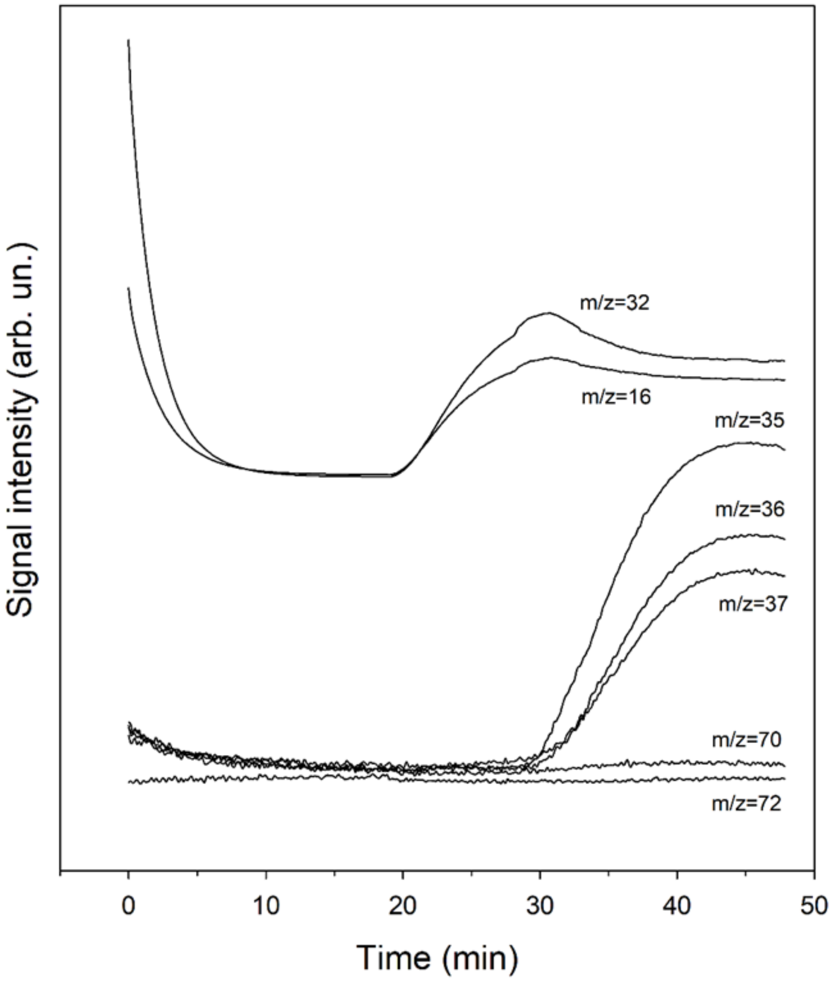

Figure S13. *Mass analysis of gases released from* ***cycled H_2O-LiCl-IL****. The intensity scale is given in arbitrary units; however, that for m/z = 35, 36, 37, 70, and 72 is logarithmic to better enhance the differences. Note that, in agreement also with MS results (Section S.2.6), no appreciable increase in Cl_2 signals (m/z = 70, 72) is observed at both room temperature and 200 °C.*

Table S3. *Retention time, name, experimental conditions (no electrolysis/electrolysis) and % match (quality of match between library spectrum and recorded one) for chemical species detected by SPME-GC/MS technique.*

t_r / min.	**Compound**	No Electrolysis	Electrolysis	% match
11.02	butanal	x		96
11.94	ethyl acetate	x	x	90
13.91	1-chlorobutane	x	x	90
19.35	*N*-methylformamide		x	86
26.42	1-methylimidazole	**x**		86
28.72	1,4-dimethylimidazole	**x**		90
30.1	*N*-butylformamide	x	x	94
30.5	5-chloro-1-methylimidazole	x	x	93
34.73	*unknown*	x		
36.02	4,5-dichloro-1-methylimidazole		**x**	94
36.65	tridecane	x	x	97
36.8	1-methoxy-4-(1-propenyl)benzene		**x**	98
37.03	*unknown*		**x**	
38.11	*unknown*	x		
38.36	tetradecane	x		98
39.06	*unknown*	x	x	
39.16	1-butyl-3-methyl-2,4,5-trioxoimidazolidine	x	x	94
39.4	*unknown*	x	x	
40.45	*unknown*		**x**	
